# Tailoring Nickel Porous Structure via Dynamic Hydrogen Bubble Template for Efficient Alkaline Hydrogen Evolution

**DOI:** 10.1021/acsomega.5c12357

**Published:** 2026-02-19

**Authors:** Gabriel G. Borges, Marina Medina, Ramiro M. dos Santos, André H. B. Dourado, Maísa A. Beluomini, Vivian V. França, Juliana F. de Brito

**Affiliations:** † Institute of Chemistry, Araraquara, Department of Analytical, Physical-Chemical and Inorganic Chemistry, 28108 São Paulo State University (UNESP), Rua Professor Francisco Degni, 55, Araraquara, São Paulo State 14800-060, Brazil; ‡ School of Agricultural and Veterinary Sciences, São Paulo State University (UNESP), Via de Acesso Prof. Paulo Donato Castellane s/n, Jaboticabal, São Paulo State 14884-900, Brazil

## Abstract

Nanoporous nickel (Ni_np_) films were synthesized via a dynamic hydrogen bubble template (DHBT) to be applied as a catalyst for the hydrogen evolution reaction (HER) in alkaline media. This research highlights the critical role of deposition parameters in controlling the structure, morphology, and catalytic activity of Ni_np_. Ti and Ni were employed as substrates to promote hydrogen bubble evolution and the nucleation and growth of homogeneous Ni_np_. The influence of deposition current density (0.5–2.0 A cm^–2^) and duration (50–300 s) on the morphology, electrochemical performance, and mechanical stability of the Ni_np_ were systematically investigated. Scanning electron microscopy (SEM) revealed that higher current densities and longer deposition times promoted pore nucleation and growth, resulting in a homogeneous Ni_np_ network with a cauliflower-like morphology. Electrochemical characterizations showed that electrodes prepared at 2.0 A cm^–2^ for 300 s exhibited the lowest overpotentials (158 ± 13.3 mV on Ti and 180 ± 37.1 mV on Ni substrates) and maintained stable current densities over 24 h of chronoamperometric testing. Electrochemical impedance spectroscopy highlighted the influence of the substrate and deposition parameters on charge transfer resistance and electrode roughness. Density functional theory calculations indicated that interstitial oxygen in the Ti substrate induces charge depletion on the surface Ti atoms, enhancing hydrogen adsorption. This work demonstrates that DHBT method offers an efficient approach for developing high-performance nanoporous Ni as electrocatalysts toward sustainable hydrogen production.

## Graphical abstract

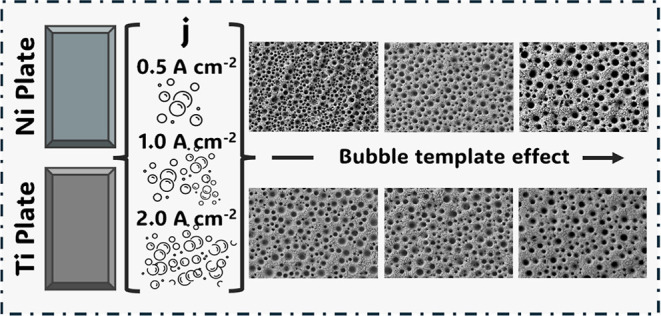

## 1 Introduction

The water electrolysis (WE) process stands out as a sustainable technology for synthesize pure and low-carbon hydrogen (H_2_).^1–3^ In turn, H_2_ is an important feedstock to NH_3_ synthesis and, recently, has been recognized as an important renewable alternative for carbon-based fuels.^4^ To split the water molecule, two fundamental processes, known as the oxygen evolution reaction (OER) and hydrogen evolution reaction (HER), occur at the anode and the cathode surfaces, respectively.^5^

In light of thermodynamics, the HER and OER occur at thermodynamic potentials of 0 and 1.23 V vs RHE, respectively, leading to an overall cell potential of 1.23 V vs RHE under standard conditions.^6–8^ Practically, the passage of electric current through the system increases the applied potential above the equilibrium value, mainly due to polarization processes.^9^ As a result, an overpotential value must be applied to overcome the kinetic limitations at the cathode and anode sides.^10^

On this point, the use of electrocatalyst materials plays a big role in reducing the activation overpotential, facilitating the electron transfer at the electrode/electrolyte interface.^11–14^ Pt-based materials are the most active catalysts for HER due to their optimal H-binding Gibbs energy, which can be accessed on the Volcano curve.^15,16^ However, the high costs associated with its scarcity limit its large-scale application to the hydrogen economy.^17^

In contrast, Ni is an abundant and low-cost element, presenting appropriate corrosion resistance and acceptable activity toward the HER in alkaline media.^18–22^

Among the reported methods for preparing high-surface-area materials, such as chemical vapor deposition, spraying, sputtering, hydrothermal, and alkaline leaching, electrochemical deposition stands out as a comparatively simpler and more cost-effective technique, providing a one-step route to prepare nanoporous , self-supported electrodes.^23^ Furthermore, the absence of a polymeric binder contributes to decreasing the electronic resistance in the substrate/catalyst interface.^24^

The dynamic hydrogen bubble template (DHBT) is a derivative of the electrodeposition method.^25,26^ Upon application of high current densities, bubbles are released on the substrate surface. The remaining ions from the deposition solution are deposited on the substrate surface between the bubbles, yielding a porous catalyst film.^27,28^

The process of using bubbles as dynamic templates excludes the post-treatment steps to remove the template, which, in turn, decreases the potential damage to the catalyst structure.^29,30^

The porous size depends on the size of the evolved bubbles, which, in turn, can be modulated by deposition parameters.^31,32^ Siwek and co-workers studied the effect of supporting electrolyte on the nanoporous characteristics and catalytic activity, showing that the electrolyte conductivity was responsible to tailor the morphology of the Ni metallic foams.^33^ Recently, porous size and distribution were evaluated as a function of current density in fixed time.^34^ Moreover, the DHBT has been used to prepare bimetallic foam-like catalysts, in an attempt to decrease the HER overpotential relying on synergistic effects.^35,36^

Trofimova and co-workers^34^ reported a porous Ni electrode exhibiting an overpotential of 338 mV at 100 mA cm^–2^, while an ultrasonically assisted DHBT-derived Ni electrode achieved 126 mV at 10 mA cm^–2^, with a Tafel slope of 150.4 mV dec^–1^ and stability of 50 h at −10 mA cm^–2^.^37^ These findings indicate that a highly porous architecture effectively enhances HER activity, although they are often accompanied by higher Tafel slopes, which are commonly attributed to mass transport limitations and hydrogen bubble accumulation within the porous network.

The choice of substrate also plays a critical role in determining the nucleation process, film growth, and adhesion, depending on their distinct physical properties such as electrical conductivity.^38,39^ In this sense, we synthesized a Ni_np_ film by the DHBT to be applied as an electrocatalyst for the HER in alkaline media. Ni and Ti were selected to be compared as substrates due to their chemical stabilities, low prices, and good activities toward the HER, which makes them a good platform for bubble release and consequent formation of a nanoporous structure.^40,41^

## 2 Materials and Methods

### 2.1 Preparation of Substrates

Metallic Ni and Ti plates (1.5 cm × 0.5 cm) were used as substrates for the electrodeposition of the materials. Initially, the plates were mechanically polished by using silicon carbide abrasive papers of different grit sizes (600, 800, 1200, 1600, and 2000) to ensure a smooth and clean surface. After polishing, the plates were ultrasonicated in methanol for 10 min for degreasing, followed by ultrasonic treatment in an aqueous 20% (v/v) HCl solution for 20 min to remove surface oxides. The plates were then thoroughly rinsed with deionized water and dried at room temperature.

### 2.2 Electrodeposition of the Nanoporous Ni Film

The electrodeposition process was carried out in a conventional three-electrode cell using the pretreated Ni and Ti plates as working electrodes. Ag/AgCl/Cl^–^_(sat. KCl)_ was used as the reference electrode, and a Ni plate (3.0 cm × 2.0 cm) served as the auxiliary electrode.

The electrolyte solution was composed of 0.1 mol L^–1^ NiCl_2_·6H_2_O, 1.0 mol L^–1^ NH_4_Cl, and 1.0 mol L^–1^ NaCl dissolved in deionized water. The solution exhibited an acidic pH value of 3.5. Before deposition, the electrolyte was purged with N_2_ for 10 min to remove dissolved oxygen, and the flux was maintained throughout the experiment.

The electrodeposition was performed via chronopotentiometry at current densities of 0.5, 1.0, and 2.0 A cm^–2^ (based on the geometric area of 0.25 cm^–2^) for deposition times of 50, 150, and 300 s. All experiments were carried out at room temperatures. The nanoporous (np) nickel film was labeled Ni_np_, in which films deposited on Ni substrates were labeled Ni_np_/Ni, and those on Ti substrates were labeled Ni_np_/Ti.

### 2.3 Physical Characterization

The surface morphology of the electrodes was examined by using a high-resolution field-emission scanning electron microscope (FEG-SEM) JEOL JSM-IT500HR). Structural characterization was performed by X-ray diffraction (XRD) using monochromatic Cu Kα radiation (λ = 1.5406 Å) in θ–2θ configuration, over a 2θ range of 10° to 130°, with a scan rate of 2° min^–1^. Analyses were conducted using a SmartLab SE diffractometer (Rigaku), equipped with a sealed 3 kW X-ray tube operating between 30–40 kV and 5–30 mA.

### 2.4 Electrochemical Characterization

Electrochemical measurements were performed in 1.0 mol L^–1^ KOH solution, using a Hg/HgO/OH^–^_(1M KOH)_ reference electrode and a graphite plate (2.5 cm × 2.0 cm) as the auxiliary electrode. All measurements were carried out under a N_2_ atmosphere at room temperature.

All Ni_np_ films were pretreated by chronoamperometry at −0.97 V for 25 min to reduce surface oxides. The HER activity was assessed by linear sweep voltammetry (LSV) from −0.4 to −1.4 V vs Hg/HgO/OH^–^_(1M KOH)_, at 5 mV s^–1^. Electrochemical impedance spectroscopy (EIS) was performed at −1.0 V, with a frequency range from 100 kHz to 50 mHz and an amplitude of 10 mV. Before EIS acquisition, −1.0 V was applied for 5 min to stabilize the double layer region at the electrode/electrolyte interface.

The stability of the prepared electrodes was evaluated by chronoamperometric experiments, at −1.25 V vs Hg/HgO/OH^–^_(1M KOH)_ applied for 24 h. All potentials were converted to the reversible hydrogen electrode (RHE) scale using eqs 1 and 2:

1 $$E_{RHE}=E_{Hg/HgO/OH-\left(1MKOH\right)}+0.098+\left(0.059\times pH\right)$$

2 $$E_{RHE}=E_{Ag/AgCl/Cl-\left(sat.KCl\right)}+0.197+\left(0.059\times pH\right)$$

### 2.5 Computational Methods and Technical Details

#### 2.5.1 Initial Configurations of Atomistic Models

The fcc and hcp structure were adopted for the Ni and Ti slabs, respectively, including five layers, totalizing 864 atoms for Ti slab and 884 atoms for Ni slab. The dimensions of the box were given at the beginning of the simulation with lattice vectors of *a* = 29.98 Å, *b* = 26.00 Å, and e *c* = 29.16 Å for the Ni electrode, and *a* = 29.16 Å, *b* = 25.25 Å, and e *c* = 29.16 Å for the Ti electrode.

It was assumed that the concentration of Ni ions near the surface is significantly high, mainly due to the process of deposition. So, it was assumed a total number of 20 Ni atoms suspended between 40 H_2_O molecules. The molecules and ions were placed on the surface with the support of the computational package PACKMOL.^42,43^

#### 2.5.2 Molecular Dynamics Simulations

Molecular dynamics simulations were performed with the Large-scale Atomic/Molecular Massively Parallel Simulator (LAMMPS) code, version 29 Aug. 2024. To obtain a realistic description of the dynamics of the atomist models, it was used a reactive force field (reaxff),^44^ including the parametrization proposed in the literature.^45,46^ First, the system was minimized with the gradient conjugate algorithm to obtain the equilibrium geometry at 0 K, including a criterion for the force minimization of 0.001. After that, the thermalization process was performed. In this step, the dynamic simulations were performed with a Nosé–Hoover thermostat for the *NVT* assembled with a temperature range of around 300 K. To achieve thermal equilibrium, 5.00 ns simulations were settled with time steps of 0.1 fs to capture chemical interactions. During the dynamics, the periodic boundary conditions were kept in the plane and reflective walls in the *z* direction.

## 3 Results and Discussion

### 3.1 Effect of Deposition Time and Current Density on the Nucleation, Growth, and Morphology of Nanoporous Nickel Films

The surface morphology of the Ni_np_ films was investigated by scanning electron microscopy (SEM). Figure 1 shows the as-synthesized films prepared on a Ti substrate under different current densities. The SEM micrographs show a clear progression in the pore structure, revealing that pore nucleation and growth are strongly influenced by the interplay between current and deposition time.

**Figure 1:**
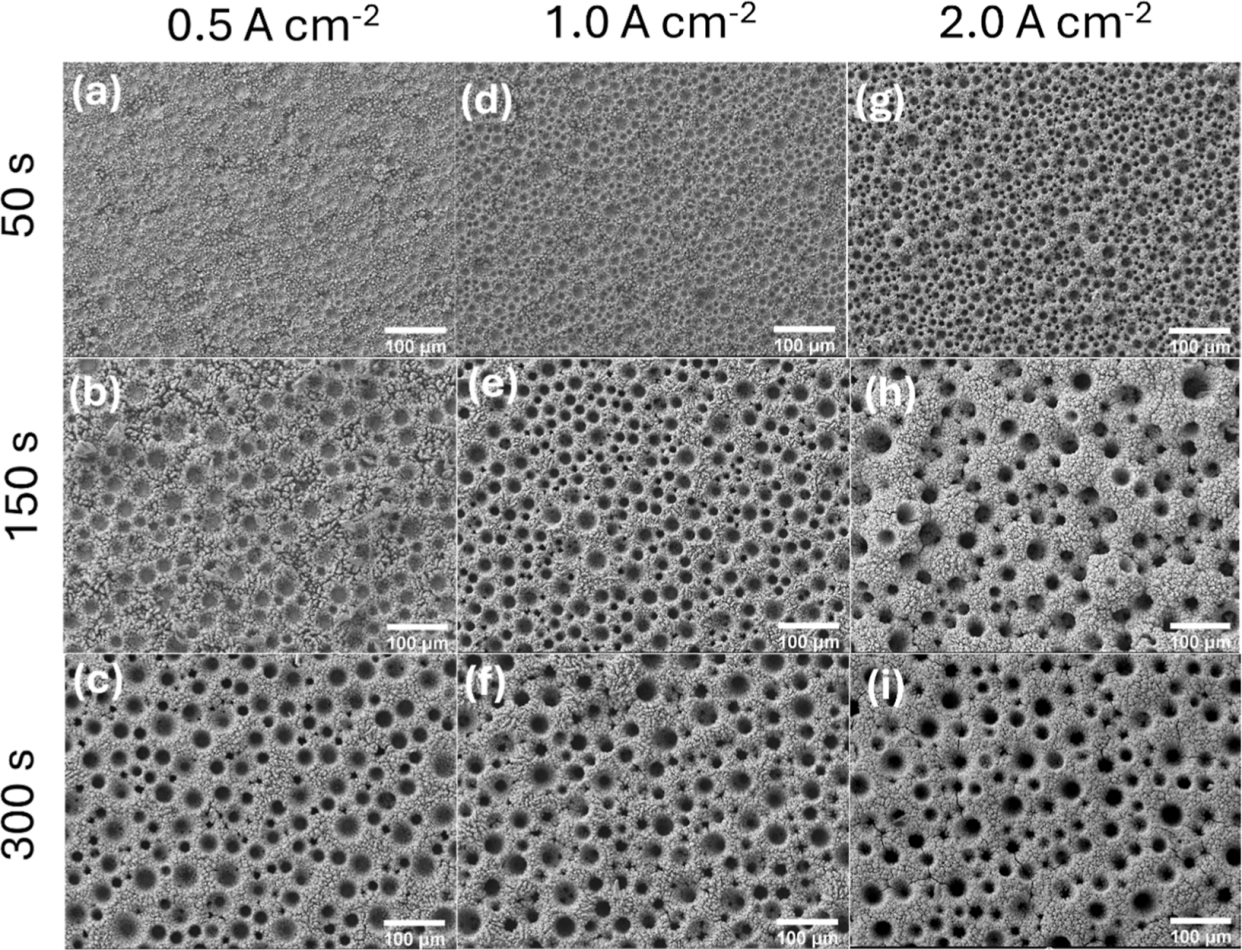
SEM images of the Ni_np_ film prepared on the Ti substrate at current densities of 0.5 (a–c), 1.0 (d–f), and 2.0 (g–i) A cm^–2^ applied during 50 s (a,d,g), 150 s (b,e,h), and 300 s (c,f,i). Magnification of 200×. Scale bar 100 μm.

The SEM images at the lowest current density of 0.5 A cm^–2^ (Figure 1a–c) reveal gradual porous nucleation on the Ti substrate. At the shorter deposition time (Figure 1a), the surface remains mostly compact with minimal pore nucleation. As time increases, a clear porous architecture emerges, with well-distributed and moderately sized pores. At 150 s (Figure 1b), the structure of the pores is a sign of a steady growth mechanism driven by controlled ion diffusion and a uniform field distribution. At the longest deposition time (Figure 1c), the pattern of the pore distribution indicates that a longer deposition time is essential in this current to achieve complete pore development.

For the intermediate current density of 1.0 A cm^–2^ (Figure 1d–f), pore formation initiates earlier. Even in a short time (Figure 1d), a fine-textured, semiporous structure is already present. With an increased time (Figure 1e,f), the porosity becomes more pronounced and interconnected. Notably, the pore diameter and depth increase with time, but signs of wall roughening and potential coalescence begin to emerge in the longest deposition (Figure 1f). This suggests that while moderate current densities accelerate pore formation, prolonged exposure may compromise uniformity due to localized field enhancement effects.

At the highest current density (Figure 1g–i), pore formation is fast. Even at the shortest deposition time (Figure 1g), the film displays a dense array of pores with a significant surface coverage. With time, the pore growth becomes increasingly heterogeneous (Figure 1h), and in the longest deposition (Figure 1i), pore coalescence is evident, and the surface exhibits textural complexity, indicative of uncontrolled growth mechanisms. These features can be related to diffusion-limited deposition at high current densities, as the process transitions into a mass- transport-controlled regime, in which the supply of electroactive species become insufficient to sustain the electrodeposition rate, resulting in an unregulated growth.^47,48^

Figure S1 shows the SEM images under high magnitudes for all synthesized electrodes, revealing the cauliflower-like morphology that remained unchanged regarding the electrodeposition parameters. Figure S1a–c,g–i reveal that the bottom region of the pores becomes darker with an increase in current density. This behavior can be associated with an increase in pore depth/film thickness because of both higher bubble release and metal deposition. This observed behavior is not as true for Figure S1d–f, following the above-mentioned nonuniform film growth. While higher current densities promote faster nucleation and growth, they also increase the risk of pore merging and loss of morphological control.

The morphological evolution of the nanoporous nickel films deposited on the Ni substrate is shown in SEM images in Figure 2a–i. Compared to Ti, the Ni substrate provides a more conductive and electrochemically compatible surface, potentially enhancing bubble evolution and film growth. This feature can be seen in Figure 2a, in which an earlier pore nucleation begins even under mild conditions of 0.5 A cm^–2^ for 50 s. At longer times of 150 s and 300 s (Figure 2b and c, respectively), a more defined network takes place, resulting in uniform and fine porosity.

**Figure 2:**
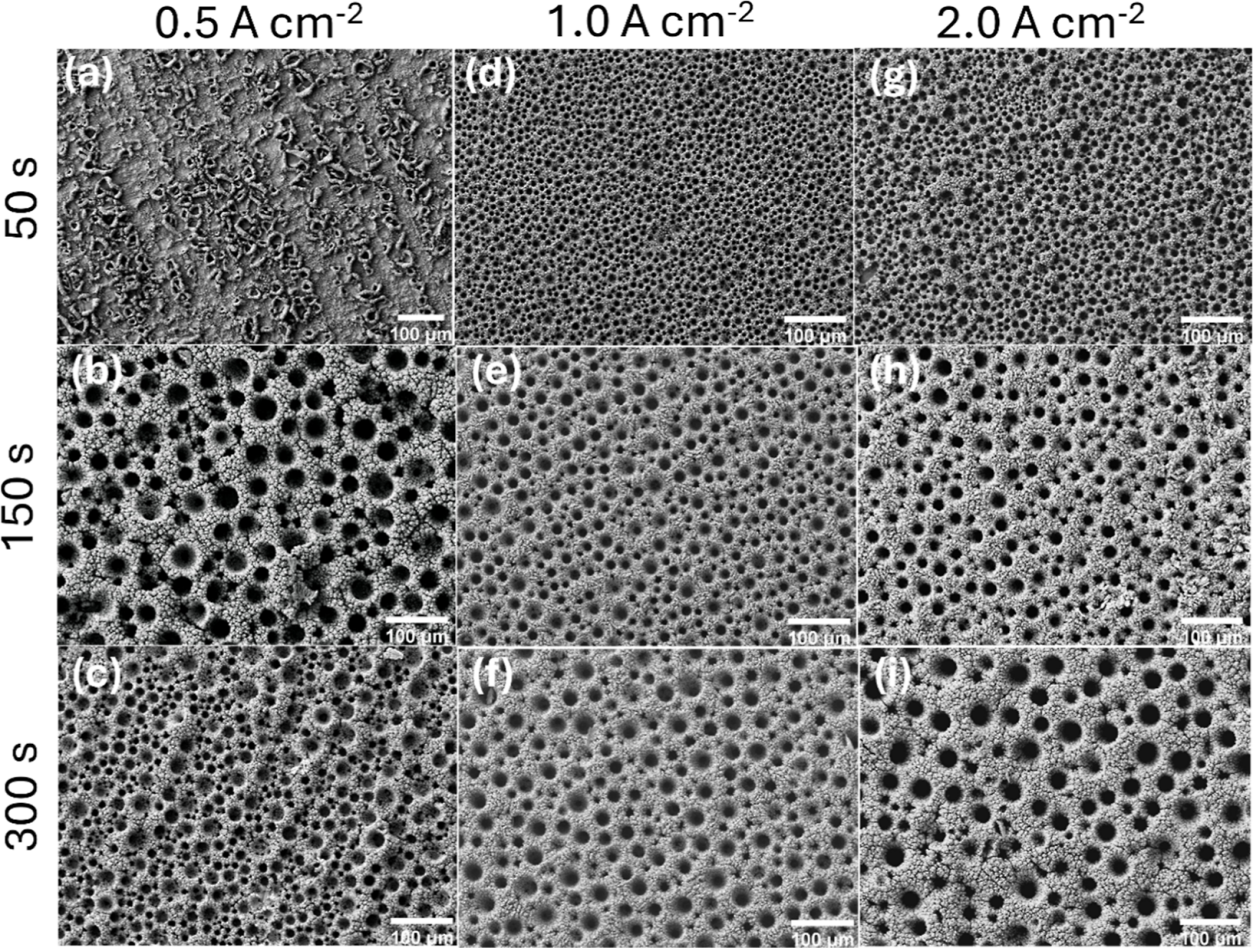
SEM images of the Ni_np_ film prepared on the Ni substrate at current densities of 0.5 (a–c), 1.0 (d–f), and 2.0 (g–i) A cm^–2^ applied during 50 s (a,d,g), 150 s (b,e,h), and 300 s (c,f,i). Magnification of 200×. Scale bar 100 μm.

At 1.0 A cm^–2^ (Figure 2d–f), the influence of the substrate was more pronounced. The morphology evolves from widely spaced, heterogeneous pores at 50 s (Figure 2d) to a highly ordered nanoporous network at 150 s (Figure 2e). At 300 s (Figure 2f), the pore seems to grow in diameter but retains structural integrity and circularity, indicating a more stable bubble evolution and consistent metal deposition.

At 2.0 A cm^–2^ and 50 s (Figure 2g), the surface is already highly porous with smaller, well-defined pores. At 150 s and 300 s, it is observed that the pores grow in diameter but maintain better regularity and structural cohesion than those on Ti. Additionally, Figure S2 shows SEM images under high magnitude for all catalysts deposited on Ni presenting the same cauliflower-like morphology for all the Ni_np_ films prepared on the Ni substrate.

The chemical composition was confirmed by the presence of Ni in the EDS analysis (Figure S3), while the XRD patterns (Figure S4) confirm the centered cubic structure showing the three characteristic diffraction facets of (111), (200), and (220).^34^

To promote a more detailed discussion, pore counting and average pore diameter calculations were performed based on SEM images acquired at a magnification of 100×. Figure 3 shows the evolution of the average pore diameter as a function of deposition time for Ni_np_ films prepared on Ti (Figure 3a) and Ni (Figure 3b) substrates. Figures S5 and S6 present the individual histograms of each case.

**Figure 3:**
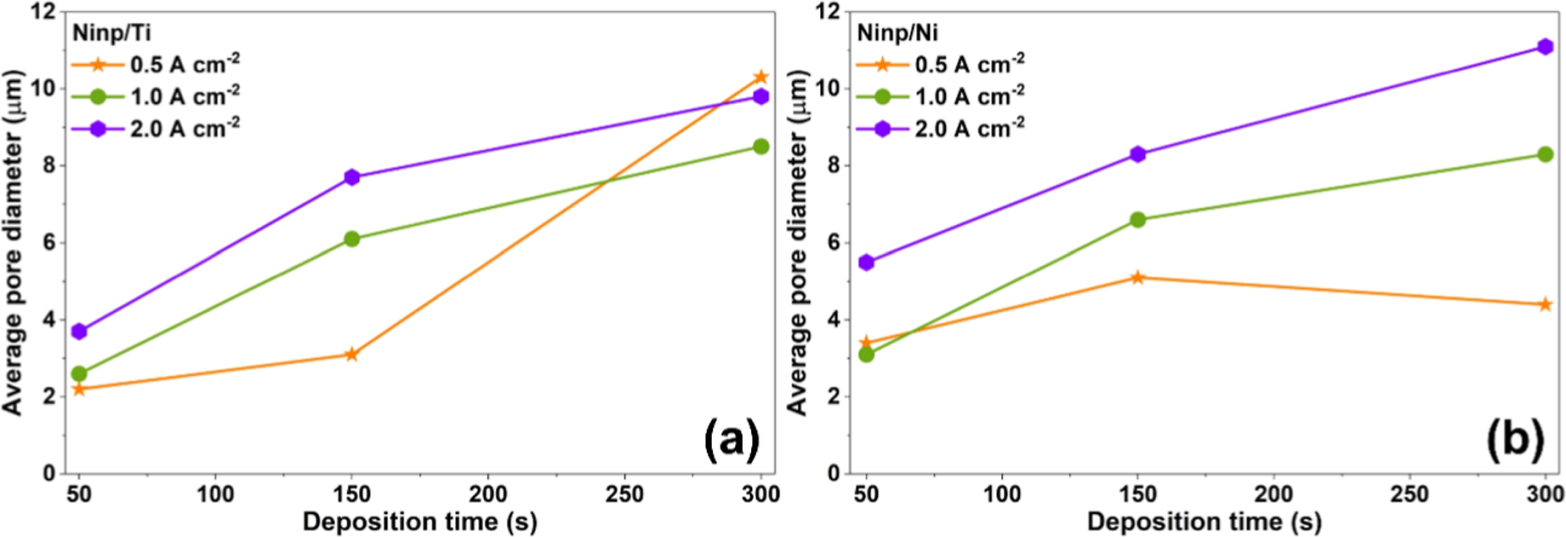
Average pore diameter obtained from pore counting analysis as a function of deposition time for Ni_np_ electrodeposited on (a) Ti and (b) Ni substrates under different applied current densities.

For the Ti substrate, Figure 3a, at 0.5 A cm^–2^, pore nucleation was initiated at 50 s with an average diameter of approximately 2.2 μm, followed by a gradual increase to 3.1 μm at 150 s and a pronounced growth at 300 s, reaching 10.3 μm. Notably, this value exceeds the average pore diameter obtained for the film synthesized at 2.0 A cm^–2^ at the same deposition time, indicating that at under low current density conditions, the deposition time plays a dominant role in pore growth. This behavior suggests that at 0.5 A cm^–2^, pore nucleation is initiated slowly but it is sustained, leading to significant pore enlargement at longer deposition times. Electrodes prepared at 1.0 and 2.0 A cm^–2^ exhibited a consistent growth pattern, with the average pore diameter increasing continuously as the deposition time increases. This indicates that, under these conditions, pore growth is favored from the early stages of electrodeposition, resulting in a more predictable morphological evolution.^49^

In contrast, for the Ni substrate, Figure 3b, the behavior at 0.5 A cm^–2^ differs markedly. The average pore diameter started at 3.4 μm at 50 s, increased to 5.1 μm at 150 s, and then decreased to 4.4 μm at 300 s. This trend suggests that although pore nucleation is promoted iniatilly, pore growth is constrained at longer deposition times, likely due to competing processes that limit the expansion of already formed pores. On the other hand, at 1.0 and 2.0 A cm^–2^, a similar behavior to that of the Ti substrate is observed, with a progressive increase in average pore diameter as the deposition time increases.^49,50^ Overall, these results indicate that intermediate and high current densities promote sustained pore growth regardless of the substrate, whereas at low current density, the influence of deposition time and substrate becomes more pronounced, leading to distinct pore evolution regimes.

### 3.2 Electrochemical Characterization

The chronoamperometric profiles obtained during the synthesis of the Ni_np_ film on Ni and Ti substrates are shown in Figure S7a–f. Regardless of the deposition parameters, it is possible to observe oscillations in the measured potential within time. As previously described by Trofimova and co-workers,^34^ the potential amplitudes are associated with the changes in the active surface area during deposition. That is, while the Ni ions are being reduced on the substrate surface, H_2_ bubbles are being released. When the bubbles block the surface, the deposition potential becomes more negative. On the other hand, the absence of bubbles increases the active surface, which, in turn, makes the potential more positive.^34^

The catalytic activities for the HER of the synthesized Ni_np_ films were analyzed through LSV. The resulting polarization curves of the Ni_np_/Ti and Ni_np_/Ni electrodes are shown in Figure 4a and b, respectively. It is possible to observe that all electrodes were active for the HER, showing an exponential increase in the current density as a function of the applied potential. Moreover, the magnitudes of the current densities were superior to those of the pristine substrate materials.

**Figure 4:**
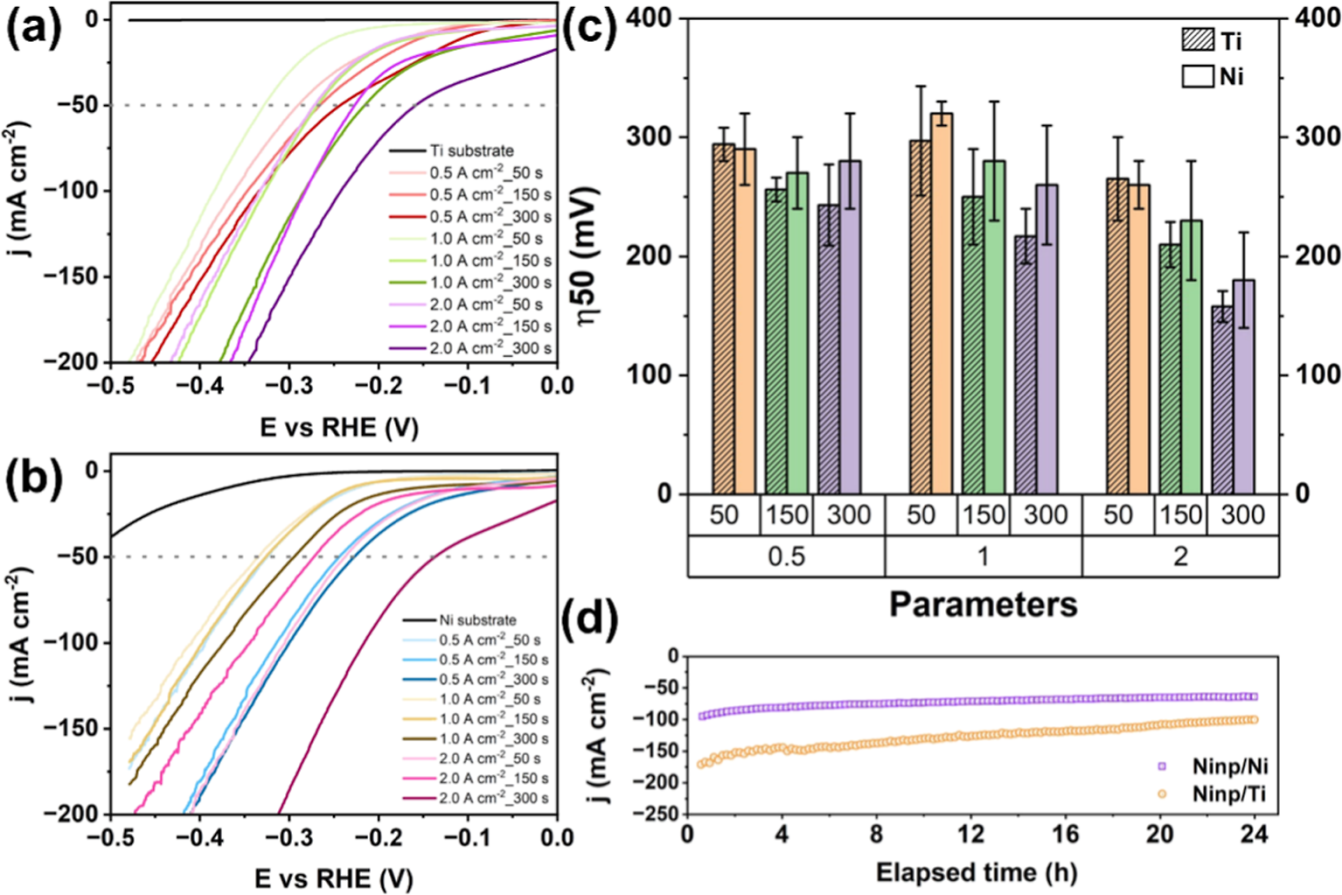
Polarization curves obtained at 5 mV s^–1^ in 1 mol L^–1^ KOH for the Ni_np_/Ti (a) and Ni_np_/Ni (b) electrodes. These curves are shown as an example of the reproducible set of measurements used to generate average data. Average overpotential values at 50 mA cm^–2^ for all the prepared electrodes in different applied current densities (0.5, 1.0, and 2.0 A cm^–2^) during 50, 150, and 300 s (c). Chronoamperometric results obtained at −350 mV vs Hg/HgO/OH^–^_(1M KOH)_ for the Ni_np_/Ni and Ni_np_/Ti electrodes prepared at 2.0 A cm^–2^ during 300 s (d).

The overpotential needed to reach a current density of 50 mA cm^–2^ (η_50_) has been used as a comparative metric to analyze the catalysts materials. Figure 4c presents the η_50_ for the prepared Ni_np_ films, which were tested in triplicate to ensure reproducibility. In general, it is possible to verify that the electrocatalytic activity improves with increasing deposition current density (0.5, 1.0, and 2.0 A cm^–2^) and deposition time (50, 150, and 300 s). Specifically, the lowest value was observed for the Ni_np_ film grown on Ti at 2.0 A cm^–2^ for 300 s. From a morphological standpoint, the Ni substrate promoted earlier pore nucleation and higher structural uniformity, consistent with its higher electrical conductivity. In contrast, the Ti substrate exhibits delayed pore nucleation at low current density but promotes significant pore enlargement at longer deposition times, yielding larger average pore diameters, which can promote HER activity due to elevated pore accessibility.

To strengthen the discussion, Table 1 presents the average values of η_50_ and the Tafel slope. Analyzing the quantitative results, it is possible to verify that the deposition condition of 2.0 A cm^–2^ for 300 s on both Ti and Ni substrates resulted in the lowest overpotential values of 158 ± 13.3 and 180 ± 37.1 mV, respectively. In contrast, the Tafel slope (*b*) presented a tendency to increase with the deposition current density and time. For example, the value for Ni_np_/Ti (0.5 A cm^–2^, 50 s) changed from 165 ± 38.2 to 303 ± 35.8 mV dec^–1^ for the Ni_np_/Ti (2.0 A cm^–2^, 300 s) electrode. Similar behavior has been reported by Harada and co-workers^51^ for fractal Ni electrodes obtained via electrodeposition, where large electrochemically active surface areas coexist with elevated Tafel slopes due to dynamic hydrogen bubble release on highly rough surfaces. This behavior suggests that the surface structure of the film changes with the growth conditions, possibly leading to the formation of thicker or more compact layers, which hinder the diffusion of reactive species.^52^ The Tafel plots used to calculate the Tafel coefficients for all the electrodes are shown in Figure S8.

**Table 1:** Reproducibility Analysis of the Overpotential at 50 mA cm^–2^ (η_50_) and Tafel Slopes (*b*) values for the Ni_np_ Film Synthesized on Ti and Ni Substrates Using Different Current Densities (*j*) and Times (*t*)

Ni_np_ deposition parameters		η_50_ (mV)		*b* (mV dec^–1^)	
*j* (A cm^–2^)	*t* (s)	Ni_np_/Ti	Ni_np_/Ni	Ni_np_/Ti	Ni_np_/Ni
0.5	50	294 ± 14.0	290 ± 29.6	165 ± 38.2	135 ± 27.2
	150	256 ± 9.74	270 ± 31.6	219 ± 30.5	151 ± 37.6
	300	243 ± 34.3	280 ± 42.9	249 ± 50.3	157 ± 54.1
1.0	50	297 ± 46.4	320 ± 13.3	142 ± 33.9	164 ± 60.7
	150	250 ± 39.6	280 ± 45.8	193 ± 40.7	211 ± 96.0
	300	217 ± 22.7	260 ± 51.9	212 ± 17.2	193 ± 56.6
2.0	50	265 ± 34.5	260 ± 24.4	152 ± 23.1	185 ± 55.0
	150	210 ± 19.5	230 ± 45.2	211 ± 48.0	184 ± 40.7
	300	158 ± 13.3	180 ± 37.1	303 ± 35.8	257 ± 4.58

It is important to highlight that the relatively large standard deviations observed for both η_50_ and *b* are consistent with the inherent characteristics of the DHBT method. That is, the stochastic nature of bubble nucleation, growth, and detachment results in nonuniform deposition and morphological heterogeneity across the film.^53,54^ Therefore, variations in pore size distribution, surface roughness, and electroactive area between samples, even under identical synthesis conditions, are common.

Another important criterion to be assessed when evaluating catalyst performance is durability. Figure 4d shows the chronoamperometric profiles of the Ni_np_/Ni and Ni_np_/Ti electrodes, prepared at 2.0 A cm^–2^ for 300 s, in which −0.3 V vs RHE was applied for 24 h. In general, both electrodes maintained a stable value of the current density within time, demonstrating effective activity and mechanical stability. Even though the Ni_np_/Ni electrode showed a more stable response within time, Ni_np_/Ti presented a higher current density value from the beginning to the end of the analysis, indicating an improved activity for the HER in the studied conditions.

Electrochemical impedance spectroscopy (EIS) was used to investigate the influence of the morphology on catalyst activity. The Bode diagram data suggest a simple capacitive time constant characteristic indicative of a capacitive element in parallel with a resistive element. This can be seen by the single bell-shaped curve presented in the Bode phase diagrams for all electrodes (Figure S9). The electrochemical circuit (EC) time constant (τ) can be related to the direct product of the resistance and capacitance of these elements.^55^ The value of such τ is defined as the inverse of this frequency of the maximum phase. Checking the Bode modulus diagram, all electrodes presented a two-plateau profile, one at high frequencies, related to the electrode/electrolyte resistance (*R*_e_), and another one at lower frequencies, related to the total resistance, or polarization resistance (*R*_p_). The resistive element observed in the EC is probably the charge transfer resistance (*R*_ct_), and its value can be obtained by the difference between *R*_p_ and *R*_e_.

Since the electrolyte is the same in all cases, the *R*_e_ values shown in Table 2 vary due to changes in the catalyst resistance. *R*_e_ remained within a narrow range, from 0.53 to 0.80 Ω cm^2^ for Ti and 0.46 to 0.68 Ω cm^2^ for Ni. In the case of the Ti current collector, all measurements resulted in a higher *R*_e_, while for the Ni, all measurements resulted in a more conductive electrode.

**Table 2:** Electrochemical Impedance Spectroscopy Parameters Obtained for Ni_np_ Films Deposited on Ti and Ni Substrates^a^

Ni_np_ deposition parameters		*R*_e_ (Ω cm_geom_^2^)		*R*_ct_ (Ω cm_geom_^2^)		*C*_dl_ (mF cm_geom_^–2^)	
*j* (A cm^–2^)	*t* (s)	Ni_np_/Ti	Ni_np_/Ni	Ni_np_/Ti	Ni_np_/Ni	Ni_np_/Ti	Ni_np_/Ni
0.5	50	0.53	0.68	62.40	74.99	0.22	0.59
0.5	150	0.69	0.68	8.41	8.91	2.25	3.54
0.5	300	0.69	0.68	12.92	6.05	1.31	7.17
1.0	50	0.63	0.46	64.24	66.70	0.22	1.22
1.0	150	0.66	0.46	7.30	50.57	2.62	4.10
1.0	300	0.80	0.46	3.63	15.68	7.00	15.33
2.0	50	0.70	0.57	18.74	6.18	4.74	5.68
2.0	150	0.72	0.57	2.82	11.55	16.80	15.14
2.0	300	0.78	0.57	1.47	1.39	44.17	32.56

a Electrolyte resistance (*R*_e_); charge transfer resistance (*R*_ct_); double-layer capacitance (*C*_dl_).

Regarding the catalyst activity, *R*_ct_ is a better evaluation parameter, since this quantity is related to the kinetic limitations of a faradaic process.^56–58^ From these parameters, it can be observed that a deposition time of 50 s was not effective for the Ni_np_ films prepared at deposition current densities of 0.5 and 1 A cm^–2^ on both Ni and Ti substrates, as indicated by *R*_ct_ values approaching 75 Ω cm^–2^. On the other hand, the lowest *R*_ct_ values were obtained for Ni_np_ films synthesized at 2 A cm^–2^ for 300 s regardless the support material, as indicated by the values of 1.47 and 1.39 Ω cm^2^ for Ti and Ni, respectively. The Ni collector presented the highest *R*_ct_ values in most cases, with exceptions for electrodes deposited at 0.5 A cm^–2^ for 300 s, and 300 s at 2.0 A cm^–2^.

The capacitance of the capacitive element can be calculated from *R*_ct_ and τ, obtained from the Bode modulus diagram and phase plots, respectively. This behavior is probably related to the electric double-layer capacitance (*C*_dl_), whose data are presented in Table 2. The electrode capacitance increased with increasing deposition current and longer times. *C*_dl_ is directly related to the electrode surface area. By performing the same procedure on a bare Ni electrode and using this value to normalize the *C*_dl_, it is possible to determine how many times each electrode exhibited a larger area (Figure S10a).

Despite the samples synthesized with 2 A cm^–2^, the Ni current collector exhibited larger electrode areas for all deposition currents with increased roughness at longer deposition times. At 2 A cm^–2^, for 150 and 300 s, the Ti current collector exhibited a higher roughness. This approach enables evaluation of the specific activity of each electrode. Accordingly, the *R*_ct_ values were refined by multiplying them by the roughness factors presented in Figure S10b. The highest values of charge transfer resistance corrected by the roughness factor (*R*_ct_^(r)^) were obtained for Ni_np_ films deposited on Ni current collector, especially those deposited at 1.0 A cm^–2^, ranging from 1835.6 to 5400.9 Ω cm^2^, followed by the film deposited at 2.0 A cm^–2^ for 150 s, 3927.9 Ω cm^2^. This indicates that these electrodes exhibited the highest kinetic barriers. The lowest *R*_ct_^(r)^ values were obtained for Ni_np_ films synthesied on Ti at 0.5 and 1 A cm^−2^.

When compared with other porous Ni electrodes obtained via DHBT-related strategies, consistent performance was observed (Table S1). Specifically, the optimized electrode of Ni_np_/Ti prepared at 2.0 A cm^–2^ during 300 s that demanded only 158 mV to reach 50 mA cm^–2^.

### 3.3 Theoretical Calculations

Since the impedance in the electrode/electrolyte interface depends on thecharacteristics of the substrate material, an atomic-scale study can be used to understand how the morphological aspects and charge states of the electrode surface contribute to the formation of H_2_ bubbles. To understand the surface characteristics, including possible point defects, it was performed molecular dynamics simulations of the Ni deposition process on Ni and Ti substrates. After that, it was carried out first-principles calculations based on density functional theory to obtain a qualitative understanding of the impact of point defects on the formation of H_2_ bubbles.

Figure 5 shows that the time needed to promote the Ni ions reduction on the Ni surface is significantly faster than that on the Ti slab. After 3.3 ns, it was observed a full adsorption of Ni and H_2_O on the Ni slab. In contrast, at the same time, it was observed partial Ni deposition on Ti, and the presence of interstitial oxygen near the surface. It is important to mention that the presence of interstitial oxygen in Ti was explored in previous theoretical and experimental results.^53,54^ Moreover, it was observed that for the Ti substrate, the deprotonation phenomenon occurs rapidly and spontaneously, with a significant number of hydrogen atoms being adsorbed on the surface, promoting the formation of hydrogen bubbles.

**Figure 5:**
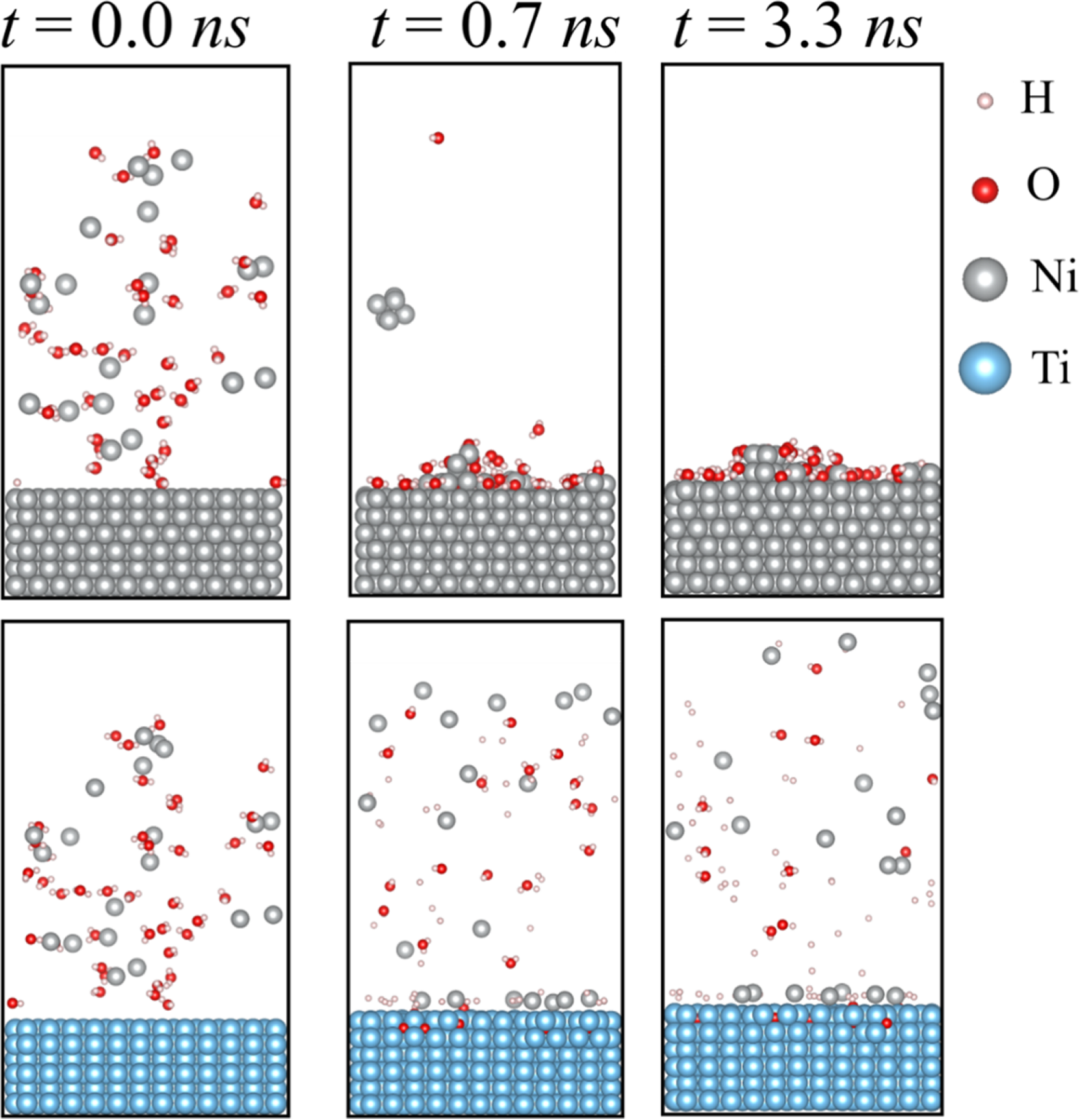
Dynamic trajectory of atomistic models. Top panel: atomistic model of Ni ions and water solution on the Ni surface. Bottom panel: adsorption of H_2_O and Ni ions on the Ti surface.

To investigate the impact of interstitial oxygen on charge transfer resistance, density functional theory calculations (DFT) implemented in the SIESTA (Spanish Initiative for Electronic Simulations with Thousands of Atoms) code,^55^ was used. Firstly, to model the Ti slab for the DFT calculations, the bulk structure with P63/pmmc symmetry containing 24 atoms of Ti was obtained from the Open Quantum Materials Database. The optimization procedure was done in iterations until each atom achieves a force lower than 0.05 eV Å^–1^. The simulation was carried out with 12 × 12 × 1 *k*-mesh. After that, from the relaxed structure, a 3 × 3 × 1 supercell was built to obtain a slab structure with 20.09 Å of the vacuum and planar lattice vectors, a and b, of 11.79 Å. All calculations for the supercell were performed including a k-mesh of 5 × 5 × 1; Perdew–Burke–Ernzerhof (PBE) exchange and correlation functional,^55^ basis double-ζ polarized (DZP), a mesh cutoff of 500 Ry, and norm-conserved pseudopotential including Troullier-Martins parametrization.^56^

After the optimization of Ti slab with interstitial oxygen, the structure maintained a total electric dipole in the *z* direction of 0.23 D. The presence of oxygen atoms in the structure required a force optimization process that incorporated spin polarization, resulting in a spin moment per atom of 2.23 μB along the *z* direction.

Figure 6 shows, on the left, the Bader charge distribution^59^ over the Ti atoms on the surface of the slab and the interstitial oxygen atom near the surface. On the right, it is possible to see that Ti atoms that interact with interstitial oxygen have a net charge depletion of +0.31*e* to +0.41*e*. At the same time, the oxygen atom keep a net charge of −1.92*e*, suggesting that most of the charge accumulated in O atoms comes from the surface. This charge depletion enhances the interaction and bonding of hydrogen on the surface through an ionic interaction, which can contribute to changing the R_ct_ value of the Ti electrode.

**Figure 6:**
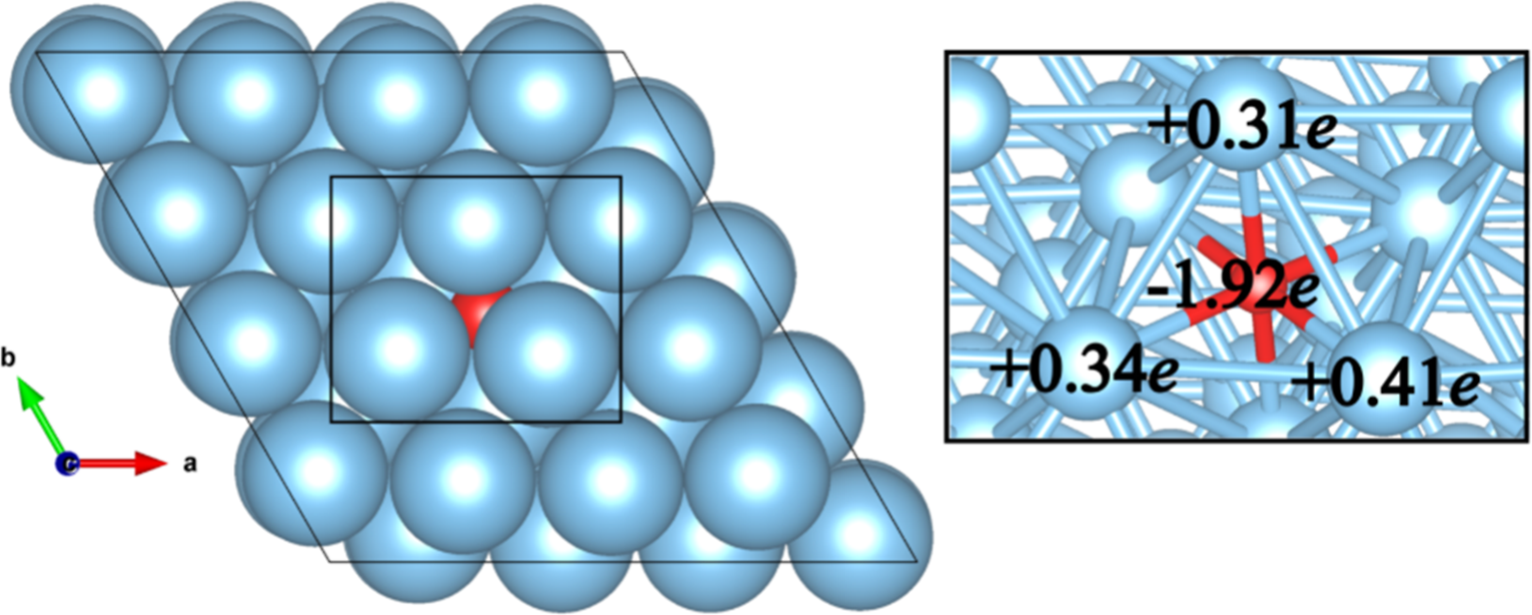
On the left, the top view of the schematic representation of the electrode with interstitial oxygen. Right, values of the Bader charge from the DFT + PBE theory level.

## 4 Conclusions

The Ni_np_ films were synthesized on both Ni and Ti substrates via the DHBT method, with superior performance observed on the Ti substrate. This behavior can be attributed to differences in film adhesion, current distribution, and mechanical stability during continuous H_2_ evolution. SEM analyses revealed that increasing current density and deposition time accelerates pore nucleation and growth for both substrates. The Ni substrate, owing to its higher electrical conductivity, promotes earlier pore nucleation and more uniform porous networks, preserving structural integrity even under high-current conditions. In contrast, the Ti substrate leads to distinct pore evolution regimes, characterized by delayed nucleation at low current density and significant pore enlargement at longer deposition times, resulting in more open and accessible pore architectures under optimized conditions. Electrochemical characterization revealed a clear improvement in HER activity with increased deposition current density and time for both substrates. However, the film prepared on a Ti substrate at 2.0 A cm^–2^ for 300 s demonstrated the best performance, with the lowest overpotential of 158 ± 13.3 mV at 50 mA cm^–2^ and the lowest *R*_ct_ of 1.47 Ω cm^_geom_2^. Despite the superior conductivity and morphological uniformity provided by the Ni substrate, the Ni_np_/Ni electrodes showed a slightly inferior HER performance, highlighting that electrical conductivity alone is not the dominant descriptor of catalytic activity. Theoretical investigations provided further mechanistic insight, showing that interstitial oxygen in the Ti substrate induces charge depletion on surface Ti atoms, strengthening hydrogen adsorption and contributing to favorable HER kinetics. This substrate-induced electronic effect compensates for the lower intrinsic conductivity of Ti and synergistically interacts with the optimized pore architecture to enhance the catalytic performance.

## Supplementary Material



## Notes

SEM images, EDS spectra, XRD diffractograms, pore size distribution histograms, chronoamperometric profiles of nickel film growth, Tafel plots, Bode phase diagrams, roughness factors calculated from the *C*_dl_ values, and a table comparing the Ni porous catalysts reported in the literature for the HER (PDF)
