# Supplementary material for: Tailoring Nickel Porous Structure via Dynamic Hydrogen Bubble Template for Efficient Alkaline Hydrogen Evolution
Source: ACS Omega. 2026 Feb 19;11(8):13865–75. doi: 10.1021/acsomega.5c12357 (PMC12961446; doi:10.1021/acsomega.5c12357)
Supplement: Supplementary file 1 [file ao5c12357_si_001.pdf]

Supporting Information

Tailoring Nickel Porous Structure via Dynamic Hydrogen Bubble Template for Efficient Alkaline Hydrogen Evolution

Gabriel G. Borges[1], Marina Medina[1], Ramiro M. dos Santos[1], André H. B. Dourado[1], Maísa A. Beluomini[2], Vivian V. França[1], and Juliana F. de Brito[1*].

[1]São Paulo State University (UNESP), Institute of Chemistry, Araraquara, Department of Analytical, Physical-Chemical and Inorganic Chemistry. Rua Professor Francisco Degni, 55, Araraquara - 14800-060, São Paulo State, Brazil.
[2]São Paulo State University (UNESP), School of Agricultural and Veterinary Sciences. Via de Acesso Prof. Paulo Donato Castellane s/n - 14884-900 - Jaboticabal, São Paulo State, Brazil

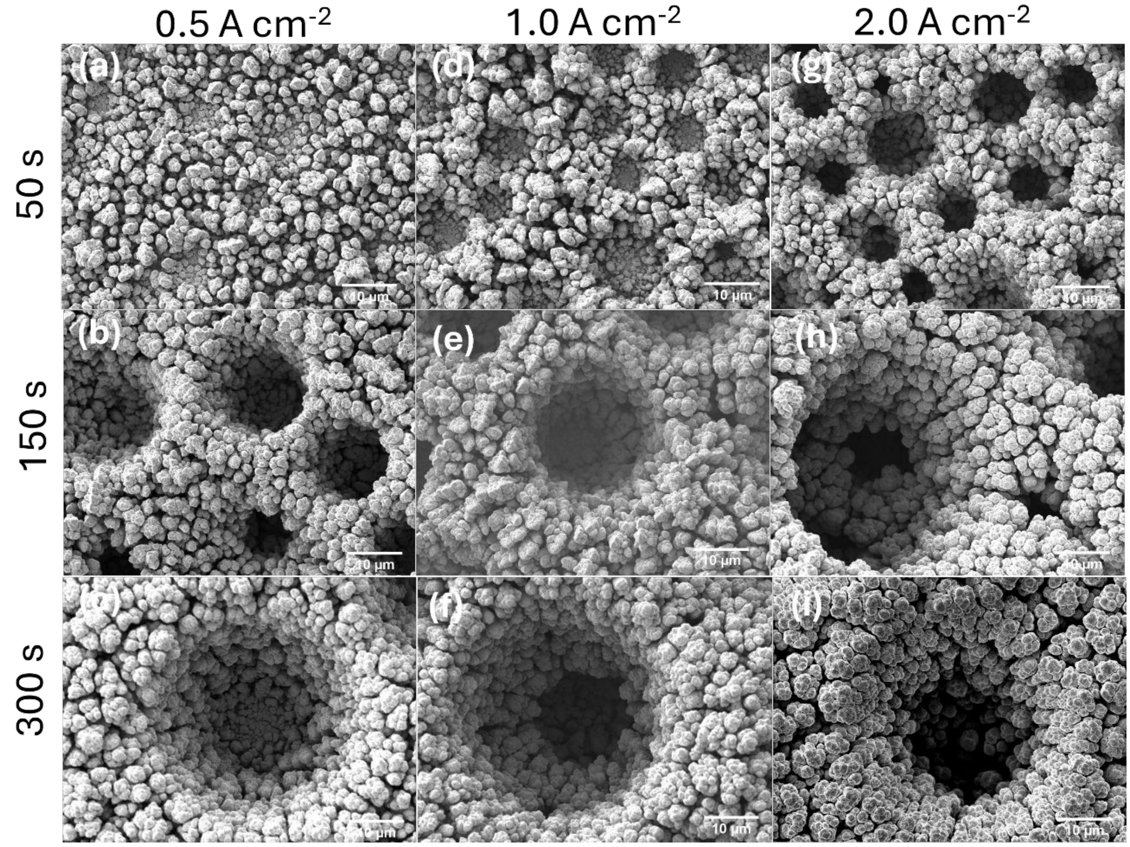

Figure S1. SEM images of Ni_{np} film prepared on Ti substrate at current densities of 0.5 (a, b, c), 1.0 (d, e, f) and 2.0 (g, h, i) A cm^{-2} applied during 50 (a, d, g), 150 (b, e, h) and 300 (c, f, i) s. Scale bar equal to 10 μm.

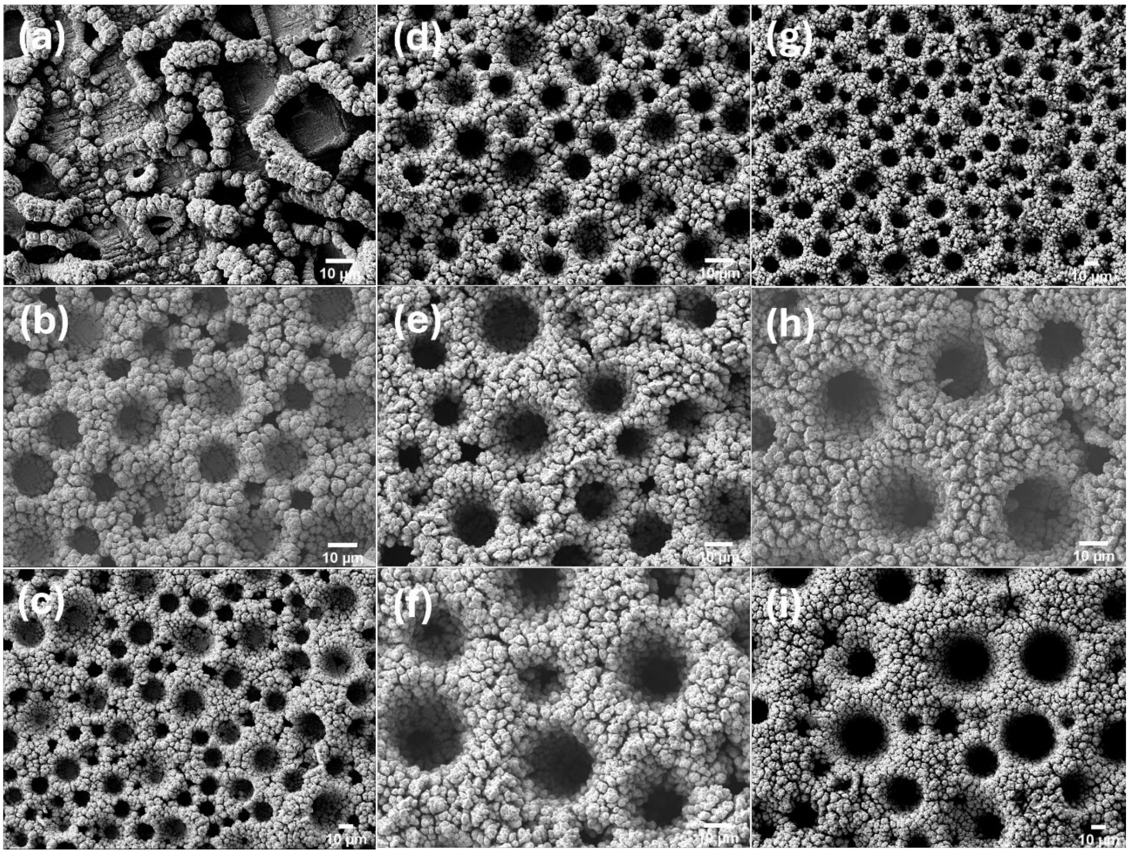

Figure S2. SEM images of Ni_{np} film prepared on Ni substrate at current densities of 0.5 (a, b, c), 1.0 (d, e, f) and 2.0 (g, h, i) A cm^{-2} applied during 50 (a, d, g), 150 (b, e, h) and 300 (c, f, i) s. Scale bar equal to 10 µm.

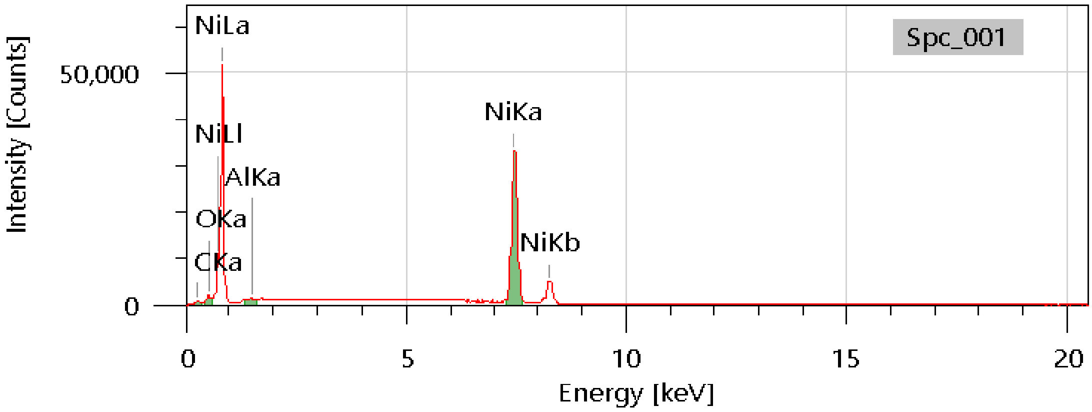

Figure S3. EDS Spectrum of the nanoporous Ni film.

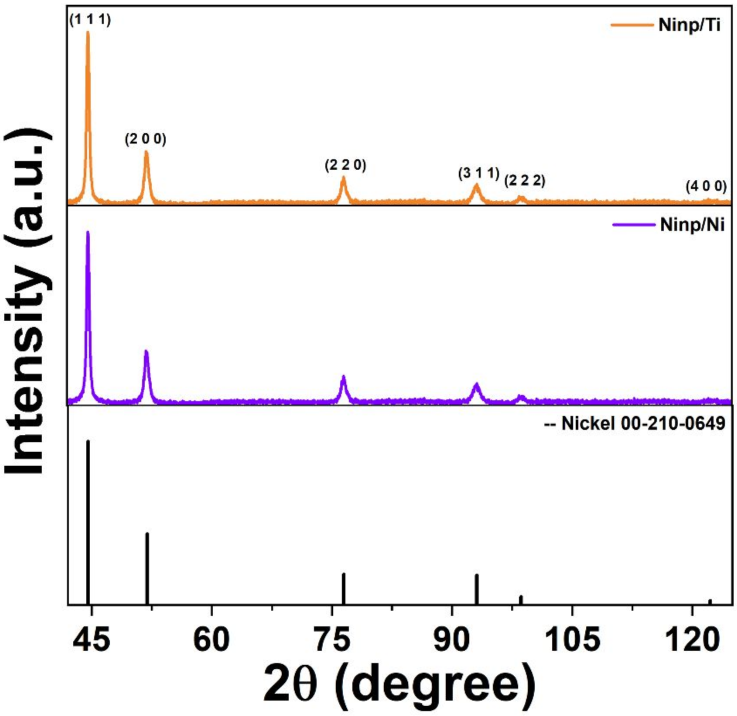

Figure S4. Diffractogram for the nanoporous Ni film.

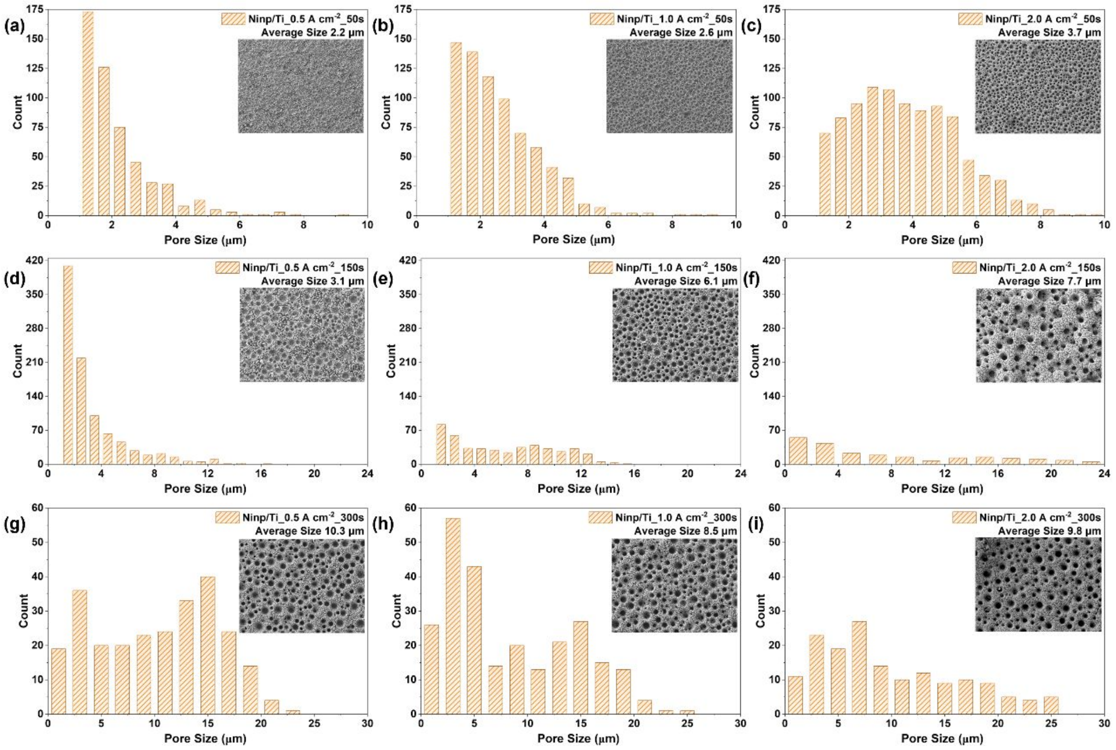

Figure S5. Pore size distribution histograms of Ni_{np} electrodes synthesized on a Ti substrate under different current densities and deposition times. Rows correspond to deposition times of 50 s (a, b, c), 150 s (d, e, f), and 300 s (g, h, i), while columns correspond to applied current densities of 0.5 A cm^{-2} (a, d, g), 1.0 A cm^{-2} (b, e, h), and 2.0 A cm^{-2} (c, f, i).

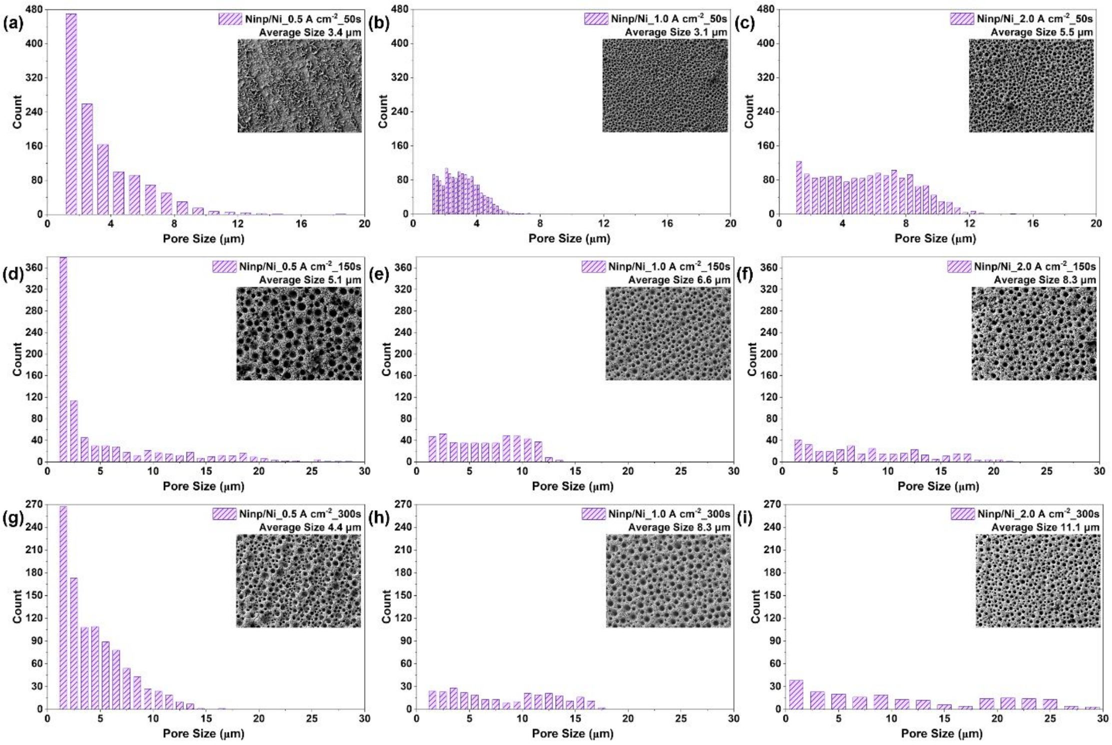

Figure S6. Pore size distribution histograms of Ni_{np} electrodes synthesized on a Ni substrate under different current densities and deposition times. Rows correspond to deposition times of 50 s (a, b, c), 150 s (d, e, f), and 300 s (g, h, i), while columns correspond to applied current densities of 0.5 A cm^{-2} (a, d, g), 1.0 A cm^{-2} (b, e, h), and 2.0 A cm^{-2} (c, f, i).

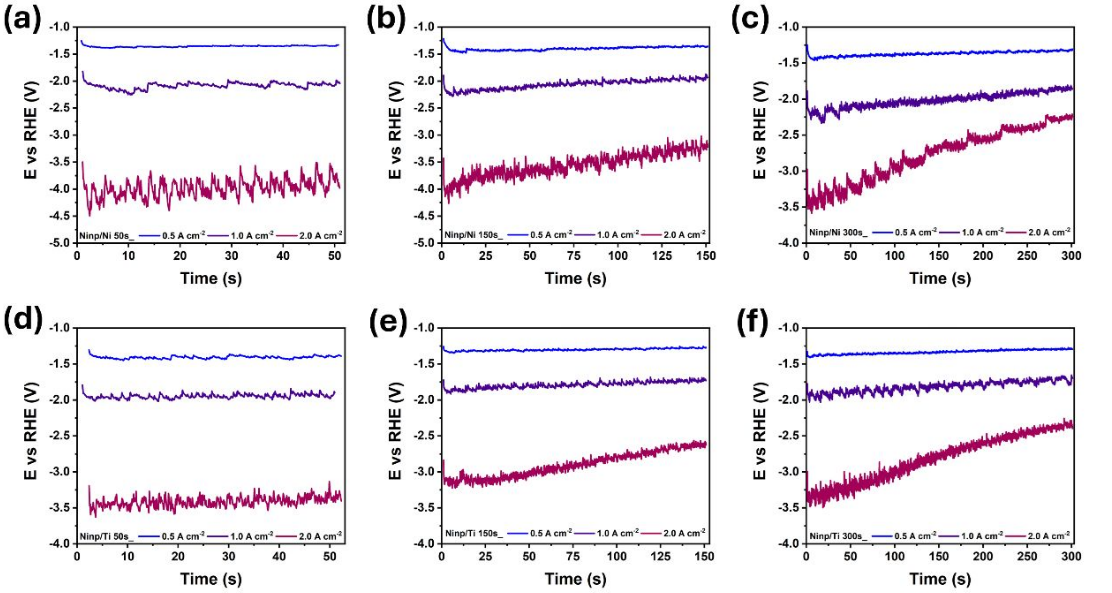

Figure S7. Chronoamperometric profiles of the nickel film growth on Ni (a-c) and Ti (d-f) substrates during 50 (a, d), 150 (b, e) and 300 (c, f) seconds for each current density of 0.5 (blue), 1.0 (dark purple) and 2.0 (dark pink) A cm^{-2}.

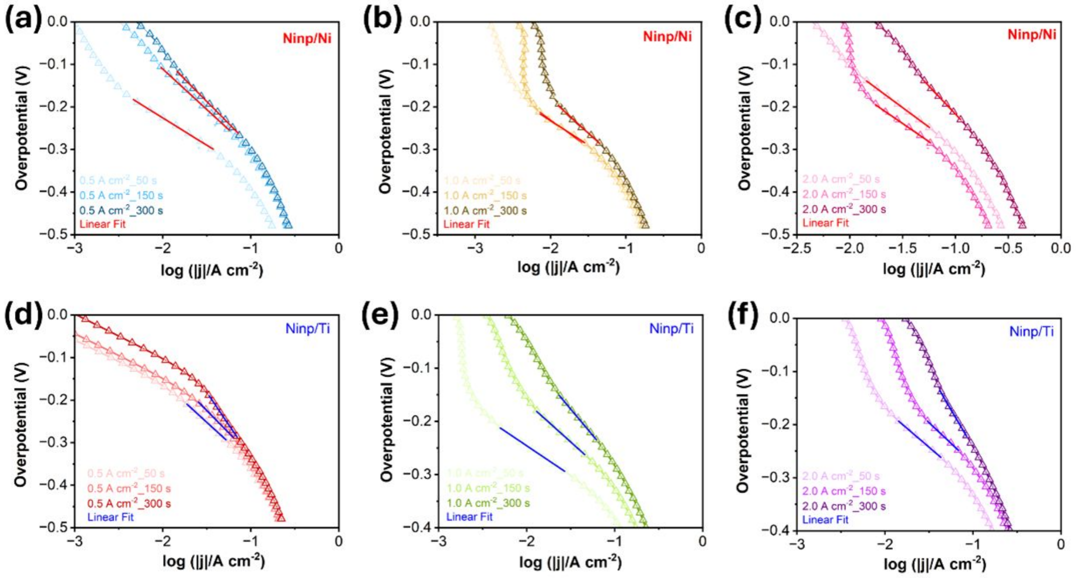

Figure S8. Tafel plots for the nanoporous Ni films prepared on Ni (a-c) and Ti (d-f) substrates.

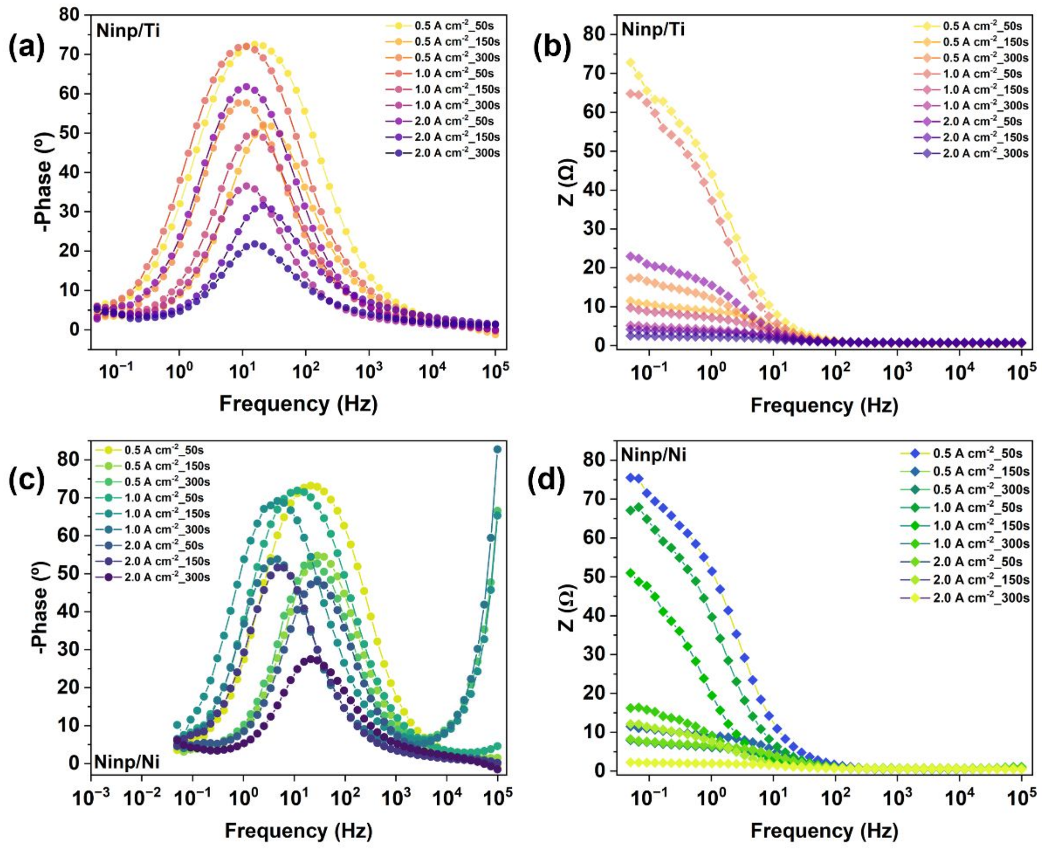

Figure S9. Bode phase diagrams for all electrodes under Ni and Ti substrates.

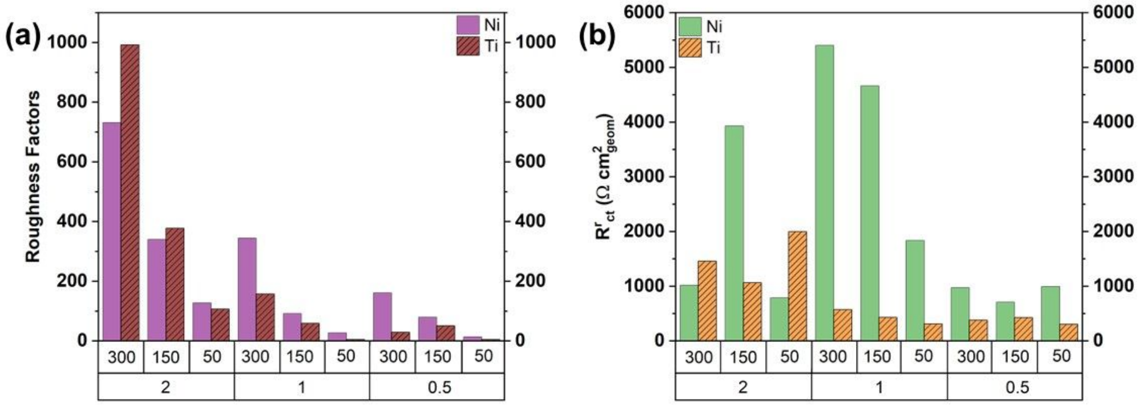

Figure S10. Roughness factors calculated from the C_{dl} values (a). Charge-transfer resistance corrected by the roughness factor ($R^{(r)}_{ct}$).

Table S1. Ni porous catalysts reported in literature and applied for hydrogen evolution reaction.

Electrode	Synthesis	η (mV)	b (mV dec^{-1})	Stability	Ref.
**Ni porous**	DHBT	338 at 100 mA cm^{-2}	107	N/A	[4]
**Ni-Co**	Electrodeposition	86.7 at 10 mA cm^{-2}	69.8	-100 mA cm^{-2} for 10 h	[1]
**Ni@NF**	PVD	100 at 10 mA cm^{-2}	88	-100 mA cm^{-2} for 45 h	[7]
**Ni**	Electrodeposition	210 at 10 mA cm^{-2}	208	-100 mA cm^{-2} for 2 h	[8]
**Ni-DAT**	Electrodeposition	65.7 at 10 mA cm^{-2}	117.6	-10 mA cm^{-2} for 100 h	[6]
**Ni_3P_Ni**	Solid-state phosphidation	116 at 10 mA cm^{-2}	59	-0.3 V for 3 h	[3]
**Ni porous**	U-DHBT	126 at 10 mA cm^{-2}	150.4	-10 mA cm^{-2} for 50 h	[5]
**Ni**	Electro-deposition	135 at 10 mA cm^{-2}	99.3	-100 mA cm^{-2} for 12 h	[9]
**Ni-Co**	DHBT	50 at 10 mA cm^{-2}	119	-10 mA cm^{-2} for 2 h	[2]
**Ni-W-Mo porous**	DHBT	236 at 50 mA cm^{-2}	129	-20 mA cm^{-2} for 180 h	[10]
**Cu-Ni@NF**	DHBT-PRED	98 at 10 mA cm^{-2}	155	-100 mA cm^{-2} for 180 h	[11]
**Ni porous**	DHBT	130 at onset	98	-0.35 V for 4 h	[12]
**Ni_{np}**	**DHBT**	**158 at 50 mA cm^{-2}**	**303**	**-0.35V for 24 h**	**This work**
